# Expansive Central Ossifying Fibroma of the Right Mandible

**DOI:** 10.7759/cureus.52863

**Published:** 2024-01-24

**Authors:** Kelsey Carey, Robert Koenigsberg, Riya Kuklani, Paul Deitrick

**Affiliations:** 1 Radiology, Temple University Hospital, Philadelphia, USA; 2 Neuroradiology, Temple University Hospital, Philadelphia, USA; 3 Pathology, Temple University Hospital, Philadelphia, USA; 4 Oral and Maxillofacial Surgery, Temple University Hospital, Philadelphia, USA

**Keywords:** benign bone neoplasm, periodontal membrane, neoplasm, facial mass, ossifying fibroma

## Abstract

This case report discusses a 28-year-old patient who presented with a large expansile lesion of the right mandible. A maxillofacial CT showed a 6.7 x 9.1 x 7.6 cm right mandibular cystic mass containing an internal matrix of ground glass bone, representing a huge odontogenic keratocyte. Upon biopsy of the lesion, the specimen consisted of non-decalcified irregular fragments of cemento-osseous material, embedded in a minimally hemorrhagic, cellular fibrous tissue stroma, suggestive of central ossifying fibroma. This case presents an ossifying fibroma that far exceeds the average size of these masses, which typically range from 1.0 to 2.5 cm at its greatest dimension. The immense size of the lesion seen in this case is rarely encountered. This case also helps to emphasize the importance of timely diagnosis and complete resection of the lesion to prevent mass recurrence and possible malignant transformation.

## Introduction

Ossifying fibromas are classified as benign bone neoplasms that can affect both the maxilla and mandible and are most often seen in middle-aged females. The average size of peripheral ossifying fibromas measures approximately 1.0 to 2.5 cm at its greatest dimension, and incomplete resection of these masses can lead to deformity and possible malignancy. Ossifying fibromas typically affect the jaw and can occasionally be aggressive and cause local destruction. Treatment often includes full excision, and once excised, they often do not recur [1]. The purpose of this case is to demonstrate the aggressive nature of these reactive lesions and the importance of timely diagnosis and full resection.

## Case presentation

**Figure 1:**
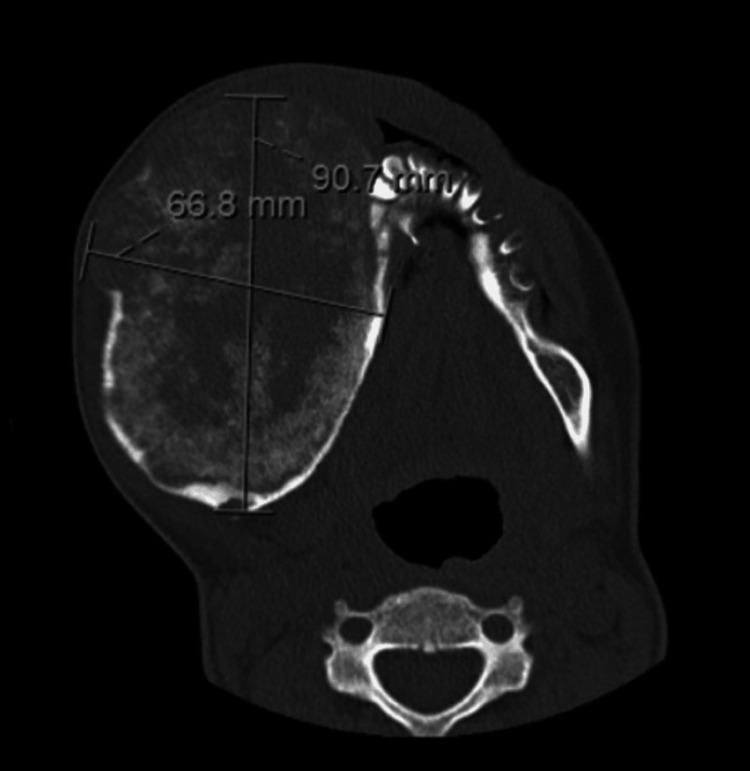
Axial CT showing the right mandibular mass measures 6.7 x 9.1 x 7.6 cm.

**Figure 2:**
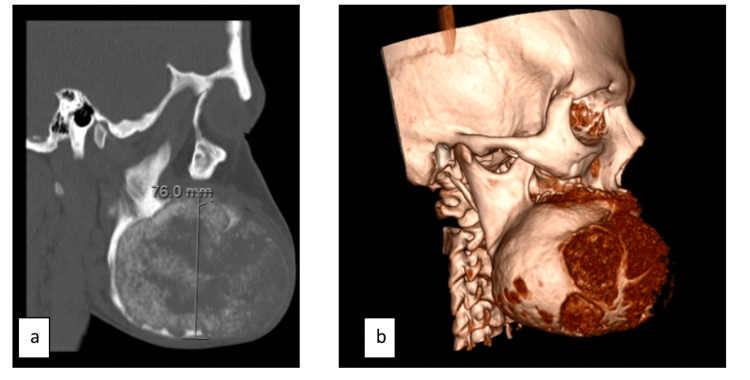
A. Depicts a sagittal CT and B. shows a sagittal reconstruction both demonstrating how the mass contains an internal ground glass calcified matrix. The posterior bony margin of the lesion extends to the anterior margin of the right mandibular ramus.

**Figure 3:**
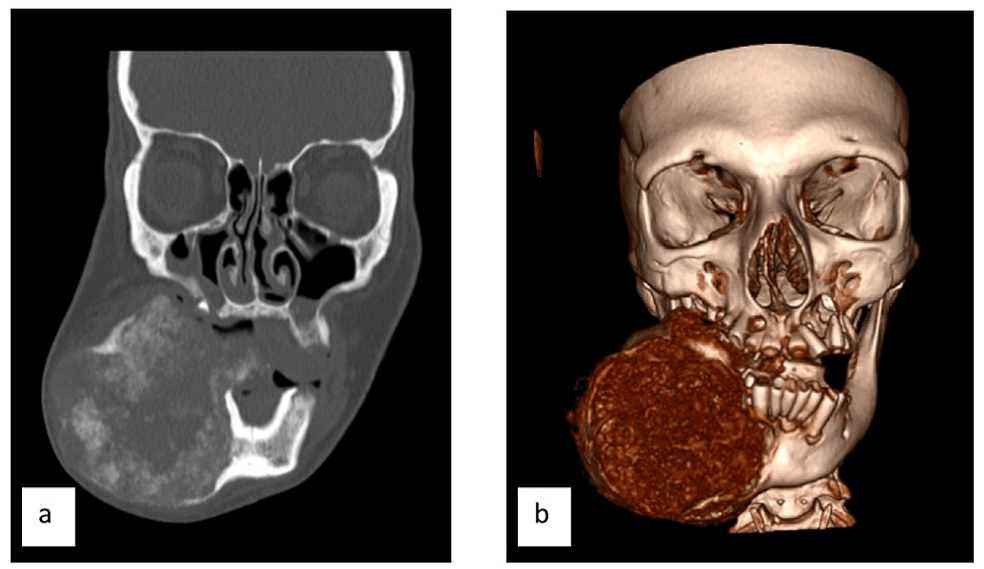
A. Depicts a coronal CT and B. shows a coronal reconstruction. Both images display thinning of the outer cortex of bone along the anterolateral margins of the mass with relative preservation of the cortex along its posterior margin.

A 28-year-old female with a past medical history of cocaine use disorder presented with a large, expansile lesion of the right mandible. The patient had no consistent follow-up prior to presenting with the mass, and it was unclear how long the patient had the mass prior to presentation. A maxillofacial CT was performed, which demonstrated a 6.7 x 9.1 x 7.6 cm complex cystic right mandibular body mass (Figure 1). The mass contained an internal ground glass calcified matrix, which was thicker both peripherally and centrally. The posterior bony margin of the lesion extended to the anterior margin of the right mandibular ramus, while medially, the mass mildly encroached on the oral cavity (Figure 2). Anteriorly, the lesion extended just adjacent to the symphysis menti. The lesion appeared devoid of teeth, with several central and left mandibular teeth identified. Due to the mass effect, the remaining central teeth were mildly rotated leftward (Figure 3). Mild bilateral mucosal thickening was identified within the ethmoids, and subtotal opacification of the right frontal and temporal sinuses was visualized. 

**Figure 4:**
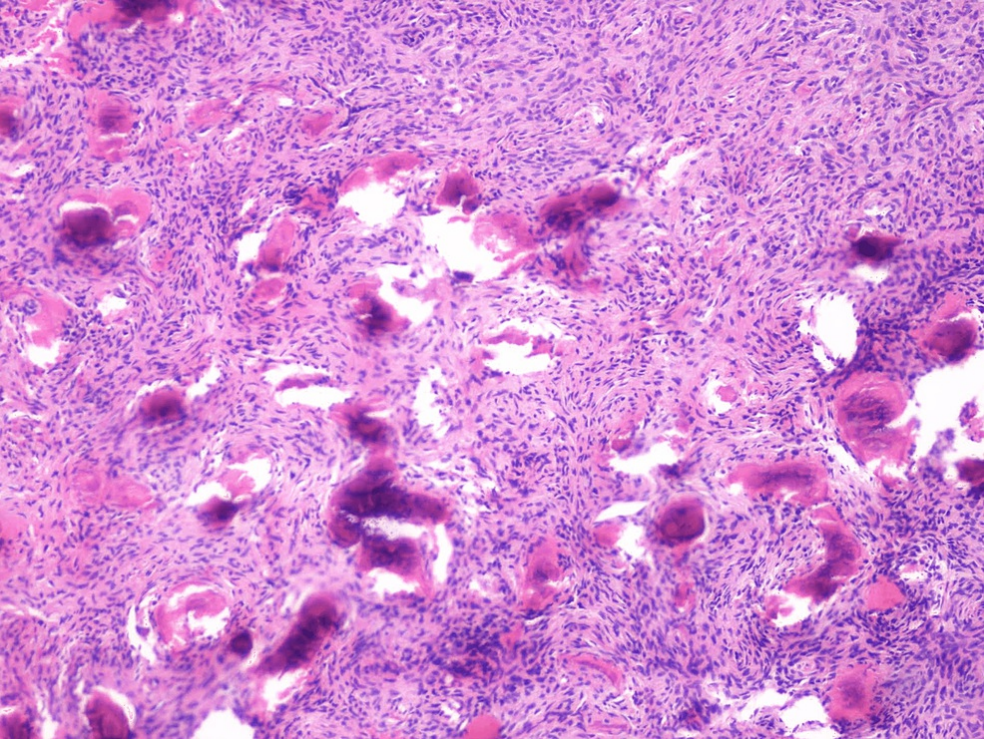
High-power photomicrograph showing a mixture of woven bone and cementum like material in a moderately cellular fibrous connective stroma (Hematoxylin & Eosin stain, 20X).

**Figure 5:**
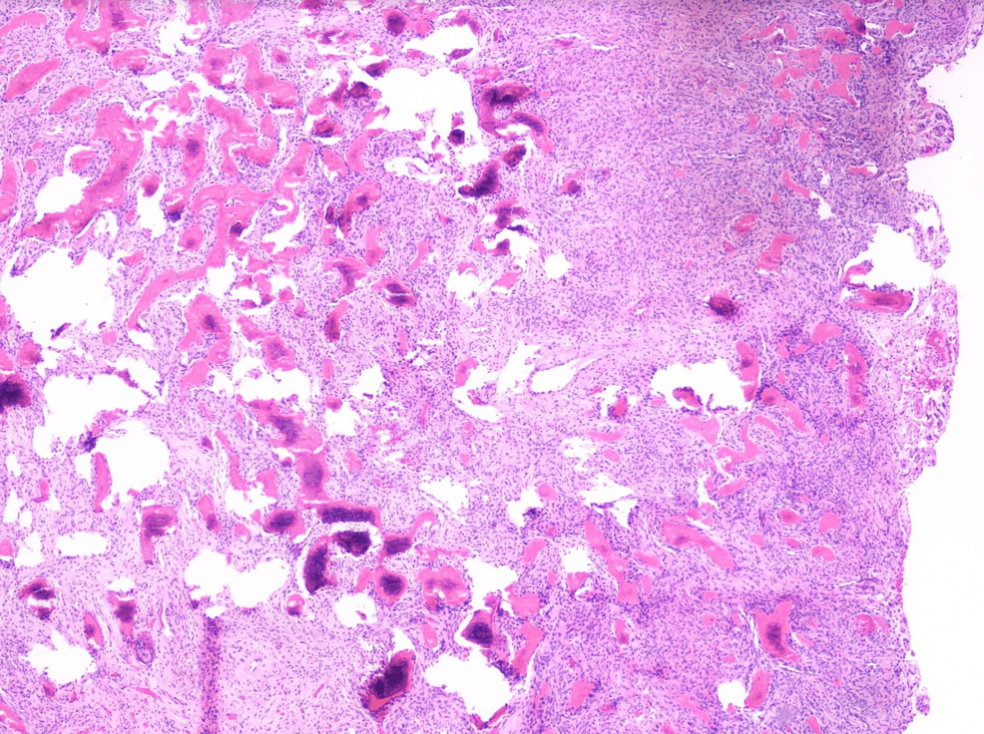
This low-magnification photomicrograph trabeculae of bone and cementum-like material in a background of cellular fibrous connective tissue(Hematoxylin & Eosin stain, 4X).

Upon biopsy of the lesion, the specimen consisted of non-decalcified irregular fragments of cemento-osseous material, embedded in a minimally hemorrhagic, cellular fibrous tissue stroma, suggestive of a central ossifying fibroma (Figures 4, 5). No evidence of malignancy was seen. The differential diagnosis included cemento-osseous dysplasia and ossifying fibroma. Clinically, cemento-osseous dysplasia does not typically expand the cortex. It consists of easily fragmented and gritty tissue that can be curetted easily from the defect but does not separate cleanly from the adjacent normal bone. In contrast, ossifying fibromas cause expansion and tend to separate cleanly from the bone, and are removed in one or several pieces. The radiographic features of an expansile lesion in the mandible, together with the microscopic findings, are suggestive of a central ossifying fibroma.

After the imaging and histopathology were evaluated, the patient underwent resection of the mass. Due to poor patient follow-up there has been limited evaluation and further treatment post-resection. 

## Discussion

Ossifying fibromas are classified as benign bone neoplasms that can affect both the maxilla and mandible. These tumors typically consist of fibrous, highly cellular tissues with varying degrees of calcified tissues that can resemble bone [1]. The average size of peripheral ossifying fibromas measures approximately 1.0 to 2.5 cm at its greatest dimension. There are four different classifications of cementum-containing lesions: fibrous dysplasia, ossifying fibroma, cementifying fibroma, and cemento-ossifying fibroma. Benign osseous fibromas are further divided into two additional categories. These are osteogenic neoplasms and non-neoplastic bone lesions [2]. Osseous fibromas are thought to originate from the periodontal membrane and most lesions often contain both cementum-like calcifications and bony material. They most often are seen in middle-aged females with the mandible being the most affected site [3]. 

Common radiographic features typically depict a cystic lesion with smooth, well-defined, corticated borders. These lesions are typically concentric with an outward expansion approximately equal in all directions. This expansion can lead to thinning and displacement of the outer cortical plate of bone. The density of the lesion is often missed, and the internal structure can contain both radiolucent and radiopaque material. As the tumor continues to expand, it can displace the teeth or the inferior alveolar canal. Aggressive growth can be observed in some lesions, which may result in massive expansile lesions involving the entire jawbone [4]. 

While the underlying cause of these masses is not fully understood, it is possible that trauma, infection, and dental extractions could contribute to the production and deposition of cementum. The treatment of osseous fibromas requires surgical removal of the mass due to the tendency of these masses to recur, and the possibility of malignant transformation. Without total resection, studies suggest recurrence often occurs between 6 months to 7 years status post resection [4]. 

## Conclusions

Ossifying fibromas are classified as benign bone neoplasms that can affect both the maxilla and mandible and are most often seen in middle-aged females. The average size of peripheral ossifying fibromas measures approximately 1.0 to 2.5 cm at its greatest dimension, and incomplete resection of these masses can lead to deformity and possible malignancy. The immense size of the lesion seen in this case is rarely encountered. It is important to be aware of the radiologic characteristics of these masses because proper diagnosis and complete resection of the lesion is critical in preventing mass recurrence and possible malignant transformation.
